# Microalgae-based biotechnology as a promising strategy for removing antibiotics from wastewater: opportunities, challenges and future directions

**DOI:** 10.3389/fbioe.2023.1248765

**Published:** 2023-08-25

**Authors:** Meng Zhang, Ruoxu Ning, Qilin Zheng, Kun Gao

**Affiliations:** ^1^ College of Biotechnology, Jiangsu University of Science and Technology, Zhenjiang, China; ^2^ Zhenjiang Zhongnong Biotechnology Co., Ltd., Zhenjiang, China

**Keywords:** microalgae, antibiotics, removal, wastewater, promising strategy, challenges

## 1 Introduction

As potentially important pharmaceuticals, antibiotics have extensively been used to improve public health, prevent infectious diseases, and promote productivity of domestic and some economical animals (Zhang et al., 2017). Every year, more than 100,000 tons of antibiotics would be consumed worldwide (Danner et al., 2019). Based on the published literatures, it can be concluded that 1) 30%–90% of antibiotics would be excreted into various environments via feces or urine because they are partially absorbed or metabolized by humans and animals (Wang et al., 2023); 2) wastewater, such as industrial (pharmaceutical industries), hospital, household, and livestock wastewater, is a sink of antibiotic residues, and a source of environmental antibiotics (Rizzo et al., 2013); 3) different concentrations of antibiotics have been detected in various wastewaters, such as industrial wastewater (26 ng L^-1^–31 mg L^-1^) (Larsson et al., 2007; Okeke et al., 2022), hospital wastewater (0.1–157 μg L^-1^) (Parida et al., 2022), and domestic wastewater (0.001–32 μg L^-1^) (Verlicchi et al., 2012); and 4) antibiotics pose a serious threat to ecological environment and human health (Wang et al., 2021). Thus, how to remove antibiotics from wastewater has attracted increasing global concern and public attention in recent years.

In conventional wastewater treatment systems, only 20%–90% of antibiotics could be removed through the process of sludge adsorption (Perini et al., 2018), and natural degradation of some antibiotics (Becker et al., 2016). In order to enhance the removal efficiency of antibiotics from wastewater, various physicochemical techniques (e.g., adsorption, flocculation, coagulation, ozonation, ion exchange, membrane filtration, electrochemical degradation, chemical oxidation, and advanced oxidation) have been developed until now (Nabilah Mohd Noor et al., 2023). Additionally, some biological methods, such as microalgae, biochars, and ligninolytic fungi, have been invented to remove antibiotics from wastewater due to their unique benefits, such as being environmentally-friendly and having low economic costs (Rambabu et al., 2020; Russell and Yost, 2021; Li et al., 2022c). Among these biological methods, microalgae-based biotechnology has attracted extensive attention recently because it possesses some merits (e.g., low cost, wastewater purification, CO_2_ sequestration, and microalgae biomass production) (Guo et al., 2016; Bhatt et al., 2022). Thus, this biotechnology is considered as an environmentally friendly strategy for removing antibiotics from wastewater and improving the quality of wastewater. However, some key challenges (e.g., low removal efficiency of antibiotics, toxicity of antibiotics and their intermediate transformation products, undefined antibiotic removal mechanisms, and effects of wastewater-born bacteria) hindering the large-scale application of this biotechnology are often not noticed in previous literatures. In this paper, opportunities, challenges and future directions about this biotechnology were described, which would help to provide new opinions into microalgae-based removal of antibiotics from wastewater, and spur researchers to carry out more investigations for obtaining practically-feasible solutions.

## 2 Opportunities, challenges and future directions

### 2.1 Opportunities of microalgae-based biotechnology used for removing antibiotics

In order to remove antibiotics from wastewater, some physicochemical methods (e.g., adsorbent adsorption, advanced oxidation, and photocatalysis) have been performed in previous studies (Mulla et al., 2023). However, the above methods have some disadvantages, which have been reviewed and listed by Leng et al. (2020) and Wang et al. (2022). Thus, novel antibiotics removal techniques need to be developed urgently according to physicochemical characteristics of wastewater.

Recently, microalgae-based biotechnology has received considerable interest in academia, and emerged as an economical, effective and green strategy for removing antibiotics from wastewater, with advantages such as effectively antibiotic and other contaminants removal, saving nutrients input, CO_2_ fixation, and potential of developing algae-derived products (Leng et al., 2020). For example, a cephalosporin antibiotic (7-amino cephalosporanic acid) could be removed effectively from wastewater by three microalgae strains (i.e., *Chlorella* sp., *Chlamydomonas* sp., and *Mychonastes* sp.) (Guo et al., 2016). It is demonstrated that the prominent removal mechanism of erythromycin by *Chlorella pyrenoidosa* is biodegradation, and some intermediate products have significant effects on the removal efficiency (Li J. et al., 2022). Therefore, this biotechnology exhibits great application opportunity and prospect in the field of antibiotic removal from wastewater.

### 2.2 Main challenges in the removal of antibiotics using microalgae

Based on previous studies, microalgae-based biotechnology has been recognized as a promising strategy for removing antibiotics from wastewater, but there is still a long way to go for large-scale application of this biotechnology due to the following limiting factors.

(1) low removal efficiency of antibiotics by microalgae. Although this technology has various advantages relative to physicochemical techniques, its overall removal efficiency is only 62.3% (Lu et al., 2023). Influential factors on the removal efficiency are structure and characteristic of antibiotics, removal capacity of microalgae, and operational conditions (e.g., hydraulic retention time, temperature, and light intensity) (Leng et al., 2020; Lu et al., 2023). Thus, low removal efficiency is the first challenge, suggesting that more investigations should be carried out for enhancing the efficiency.
(2) toxicity of antibiotics and their intermediate transformation products (TPs). The algal growth is significantly inhibited by antibiotics because they could affect the synthesis of chemicals and the activities of enzymes in algal cells (Bashir and Cho, 2016). Moreover, intermediate TPs of antibiotics possess greater toxicity than themselves (Yu et al., 2022). For example, some TPs have been identified during the biodegradation of sulfamerazine, sulfamethoxazole, and sulfamonomethoxine by microalgae, and one of them (TP126) has been proved to have high acute and chronic toxicity to green algae concurrently (Kiki et al., 2020). Thus, the toxicity of antibiotics and TPs is the second challenge, which should be paid more attention in the future.
(3) undefined antibiotic removal mechanisms. Recently, several removal mechanisms (e.g., bioadsorption, bioaccumulation, and biodegradation) have been demonstrated when microalgae are used to remove antibiotics from wastewater (Leng et al., 2020; Xiong et al., 2021). However, an explicit mechanism has not been proposed in previous investigations because different mechanisms have different contributions to the removal of antibiotics even though the same microalgae strain is used (Kiki et al., 2020; Li J. et al., 2022). Thus, exact mechanisms of interaction between microalgae and antibiotics are also a challenge when microalgae are used to remove antibiotics from wastewater.
(4) effects of wastewater-born bacteria. Many pathogenic and antibiotic-resistant bacteria, such as *Enterobacteriaceae*, *Pseudomonas aeruginosa*, *Escherichia coli*, and *Acinetobacter* sp., are detected in wastewater (Li et al., 2022b). They could influence the removal efficiency of antibiotics because interactions between microalgae and bacteria cover a wide range of relationships from cooperation to competition (Xiong et al., 2021). It has been reported that both microalgae and bacteria can serve as biosorbents for the biosorption of antibiotics in wastewater (Li et al., 2022c); however, presence of these bacteria would make the algal biomass unable to become a high-quality raw material for producing food, feed, fertilizers, cosmetics, pharmaceuticals, and nutraceuticals. Thus, how to balance the relationships between microalgae and bacteria is a challenge when microalgae-based biotechnology is used for removing antibiotics from wastewater.

### 2.3 Opinions on the future directions

In order to overcome the above challenges, more investigations should be carried out in following opinions.

(1) isolation, selection and breeding of microalgae strains. Except for the most common techniques (e.g., chemical mutagenesis, physical mutagenesis, and adaptive laboratory evolution), molecular-based approaches (e.g., heterologous transformation, homologous recombination, and gene editing tools) should also be employed individually or combined with the common techniques for generating microalgae strains with high removal efficiency and stress resilience of antibiotics (Sproles et al., 2021; Jebali et al., 2022).
(2) improvement of operational conditions of the removal strategy. In order to enhance removal efficiency of antibiotics by microalgae, concentrations and classes of antibiotics should be analyzed firstly, and then environmental and operational parameters (e.g., light conditions, hydraulic retention time, temperature, and culture’s pH) need to be optimized and improved (Bhatt et al., 2022; Wang et al., 2022). It will be always a research topic in the future due to unique properties of algal species and enormous kinds of antibiotics.
(3) deciphering antibiotic removal mechanisms by microalgae *via* omics technologies. Understanding the precise antibiotic removal mechanisms could help to develop some novel antibiotic removal techniques, and obtain more information about their toxicity (Kiki et al., 2020; Wang et al., 2022). Besides of biochemical and microbial analysis, omics technologies have been recognized as promising tools for deciphering antibiotic removal mechanisms, which would be the hotspots of future studies (Xiong et al., 2021).
(4) development of promising microalgae-based wastewater treatment process. To avoid the influences of wastewater-born bacteria, wastewater should be treated with ultraviolet irradiation, H_2_O_2_, and/or NaClO before applying the microalgae-based biotechnology to remove antibiotics from wastewater (Yu et al., 2022). In addition, novel antibiotics removal systems and promising advanced techniques will be a research topic in the future. For example, this removal strategy could be synergized with other technologies, such as advanced oxidation processes, activated sludge, and membrane filtration (Yu et al., 2022; Lu et al., 2023). Thus, promising microalgae-based wastewater treatment process should be developed further.

## 3 Summary and recommendations

**FIGURE 1:**
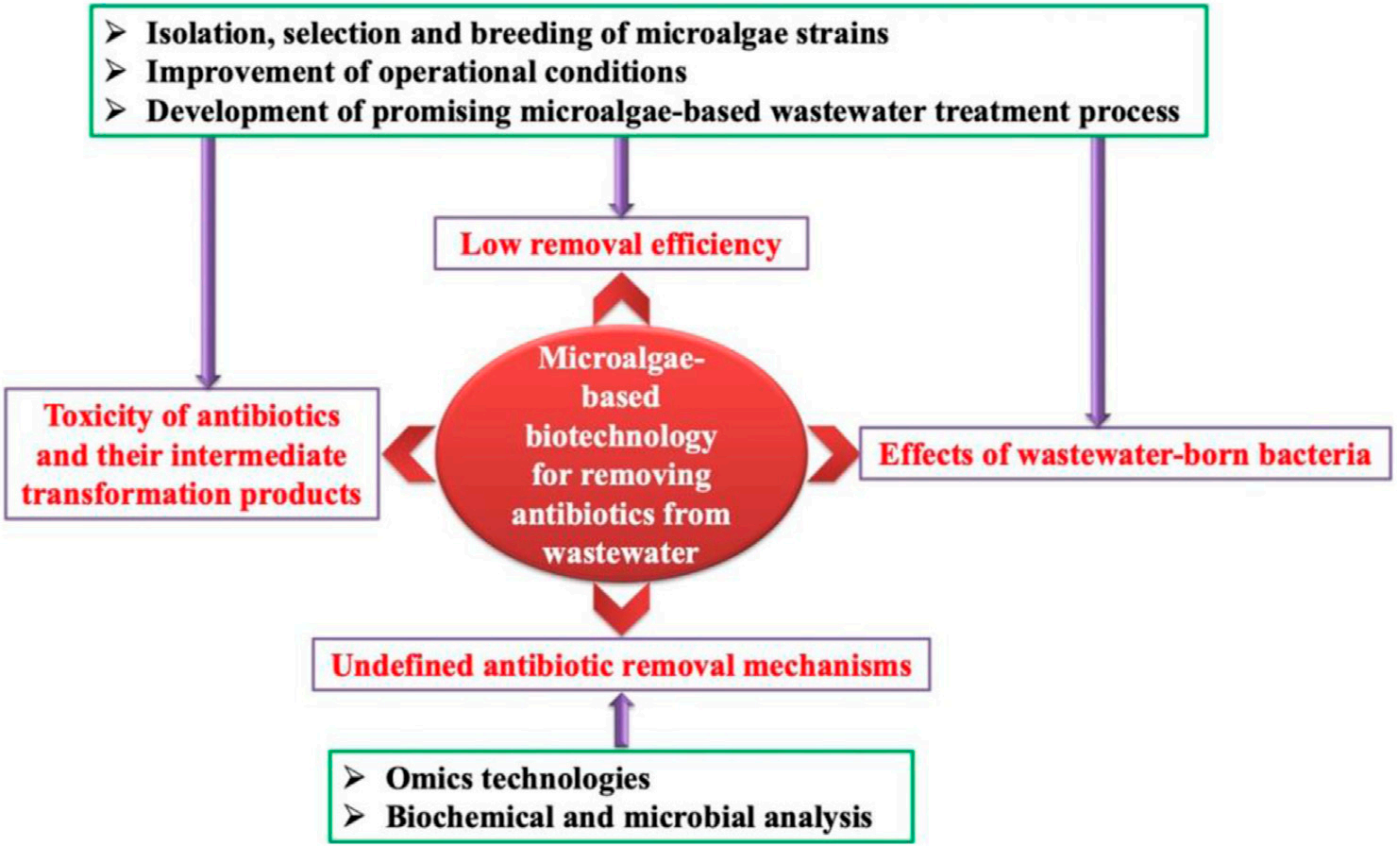
Challenges and potential solutions of using microalgae-based biotechnology to remove antibiotics from wastewater.

Nowadays, microalgae-based biotechnology has been considered as a promising strategy, and shows great potential applications in the field of antibiotic removal from wastewater. On the basis of published literatures, there are some challenges that hinder the rapid development of this biotechnology (Figure 1). Firstly, overall removal efficiency of antibiotics by microalgae is not very high, which should be enhanced further by using different techniques, such as selection of microalgae strains, optimization of operational parameters, and design of novel removal systems. Secondly, toxicity of antibiotics and the intermediate products should be paid more attention in future studies because it plays an important role during the removal of antibiotics by microalgae. Thirdly, limited information is known about antibiotic removal mechanisms by microalgae, which should be further investigated by using biochemical and microbial analysis combined with omics technologies. Finally, effects of wastewater-born bacteria should be taken seriously, and eliminated with some promising microalgae-based wastewater treatment process. Therefore, microalgae-based removal of antibiotics from wastewater has not been applied widely because of the above challenges, which should be paid more attention in the future.

## References

[B1] Bashir K. M. Cho M. G. (2016). The effect of kanamycin and tetracycline on growth and photosynthetic activity of two chlorophyte algae. Biomed. Res. Int. 2016, 1–8. 10.1155/2016/5656304 PMC5055999 27747232

[B2] Becker D. Varela Della Giustina S. Rodriguez-Mozaz S. Schoevaart R. Barceló D. de Cazes M. (2016). Removal of antibiotics in wastewater by enzymatic treatment with fungal laccase - degradation of compounds does not always eliminate toxicity. Bioresour. Technol. 219, 500–509. 10.1016/j.biortech.2016.08.004 27521787

[B3] Bhatt P. Bhandari G. Bhatt K. Simsek H. (2022). Microalgae-based removal of pollutants from wastewaters: occurrence, toxicity and circular economy. Chemosphere 306, 135576. 10.1016/j.chemosphere.2022.135576 35803375

[B4] Danner M. C. Robertson A. Behrends V. Reiss J. (2019). Antibiotic pollution in surface fresh waters: occurrence and effects. Sci. Total Environ. 664, 793–804. 10.1016/j.scitotenv.2019.01.406 30763859

[B5] Guo W. Q. Zheng H. S. Li S. Du J. S. Feng X. C. Yin R. L. (2016). Removal of cephalosporin antibiotics 7-ACA from wastewater during the cultivation of lipid-accumulating microalgae. Bioresour. Technol. 221, 284–290. 10.1016/j.biortech.2016.09.036 27643737

[B6] Jebali A. Sanchez M. R. Hanschen E. R. Starkenburg S. R. Corcoran A. A. (2022). Trait drift in microalgae and applications for strain improvement. Biotechnol. Adv. 60, 108034. 10.1016/j.biotechadv.2022.108034 36089253

[B7] Kiki C. Rashid A. Wang Y. Li Y. Zeng Q. Yu C. P. (2020). Dissipation of antibiotics by microalgae: kinetics, identification of transformation products and pathways. J. Hazard. Mater. 387, 121985. 10.1016/j.jhazmat.2019.121985 31911384

[B8] Larsson D. G. de Pedro C. Paxeus N. (2007). Effluent from drug manufactures contains extremely high levels of pharmaceuticals. J. Hazard. Mater. 148, 751–755. 10.1016/j.jhazmat.2007.07.008 17706342

[B9] Leng L. Wei L. Xiong Q. Xu S. Li W. Lv S. (2020). Use of microalgae based technology for the removal of antibiotics from wastewater: A review. Chemosphere 238, 124680. 10.1016/j.chemosphere.2019.124680 31545213

[B10] Li J. Liu K. Li W. Zhang M. Li P. Han J. (2022a). Removal mechanisms of erythromycin by microalgae *Chlorella pyrenoidosa* and toxicity assessment during the treatment process. Sci. Total Environ. 848, 157777. 10.1016/j.scitotenv.2022.157777 35926608

[B11] Li S. Ondon B. S. Ho S. H. Jiang J. Li F. (2022b). Antibiotic resistant bacteria and genes in wastewater treatment plants: from occurrence to treatment strategies. Sci. Total Environ. 838, 156544. 10.1016/j.scitotenv.2022.156544 35679932

[B12] Li S. Show P. L. Ngo H. H. Ho S. H. (2022c). Algae-mediated antibiotic wastewater treatment: A critical review. Environ. Sci. Ecotechnol. 9, 100145. 10.1016/j.ese.2022.100145 36157853 PMC9488067

[B13] Lu W. Xu C. Liu F. Su M. Cheng S. Zhang Y. (2023). Antibiotic removal efficiency by microalgae: A systematic analysis combined with meta-analysis. Process Saf. Environ. 174, 912–920. 10.1016/j.psep.2023.05.001

[B14] Mulla S. I. Bagewadi Z. K. Faniband B. Bilal M. Chae J. C. Bankole P. O. (2023). Various strategies applied for the removal of emerging micropollutant sulfamethazine: A systematic review. Environ. Sci. Pollut. Res. Int. 30, 71599–71613. 10.1007/s11356-021-14259-w 33948844

[B15] Nabilah Mohd Noor N. Hazirah Kamaruzaman N. Al-Gheethi A. Maya Saphira Radin Mohamed R. Hossain M. S. (2023). Degradation of antibiotics in aquaculture wastewater by bio-nanoparticles: A critical review. Ain Shams Eng. J. 14, 101981. 10.1016/j.asej.2022.101981

[B16] Okeke E. S. Ezeorba T. P. C. Okoye C. O. Chen Y. Mao G. Feng W. (2022). Environmental and health impact of unrecovered API from pharmaceutical manufacturing wastes: A review of contemporary treatment, recycling and management strategies. Sustain. Chem. Pharm. 30, 100865. 10.1016/j.scp.2022.100865

[B17] Parida V. K. Sikarwar D. Majumder A. Gupta A. K. (2022). An assessment of hospital wastewater and biomedical waste generation, existing legislations, risk assessment, treatment processes, and scenario during COVID-19. J. Environ. Manage. 308, 114609. 10.1016/j.jenvman.2022.114609 35101807 PMC8789570

[B18] Perini J. A. L. Tonetti A. L. Vidal C. Montagner C. C. Nogueira R. F. P. (2018). Simultaneous degradation of ciprofloxacin, amoxicillin, sulfathiazole and sulfamethazine, and disinfection of hospital effluent after biological treatment via photo-Fenton process under ultraviolet germicidal irradiation. Appl. Catal. B Environ. 224, 761–771. 10.1016/j.apcatb.2017.11.021

[B19] Rambabu K. Banat F. Pham Q. M. Ho S. H. Ren N. Q. Show P. L. (2020). Biological remediation of acid mine drainage: review of past trends and current outlook. Environ. Sci. Ecotechnol. 2, 100024. 10.1016/j.ese.2020.100024 36160925 PMC9488087

[B20] Rizzo L. Manaia C. Merlin C. Schwartz T. Dagot C. Ploy M. C. (2013). Urban wastewater treatment plants as hotspots for antibiotic resistant bacteria and genes spread into the environment: A review. Sci. Total Environ. 447, 345–360. 10.1016/j.scitotenv.2013.01.032 23396083

[B21] Russell J. N. Yost C. K. (2021). Alternative, environmentally conscious approaches for removing antibiotics from wastewater treatment systems. Chemosphere 263, 128177. 10.1016/j.chemosphere.2020.128177 33297145

[B22] Sproles A. E. Fields F. J. Smalley T. N. Le C. H. Badary A. Mayfield S. P. (2021). Recent advancements in the genetic engineering of microalgae. Algal Res. 53, 102158. 10.1016/j.algal.2020.102158

[B23] Verlicchi P. Al Aukidy M. Zambello E. (2012). Occurrence of pharmaceutical compounds in urban wastewater: removal, mass load and environmental risk after a secondary treatment-a review. Sci. Total Environ. 429, 123–155. 10.1016/j.scitotenv.2012.04.028 22583809

[B24] Wang C. Liu X. Yang Y. Wang Z. (2021). Antibiotic and antibiotic resistance genes in freshwater aquaculture ponds in China: A meta-analysis and assessment. J. Clean. Prod. 329, 129719. 10.1016/j.jclepro.2021.129719

[B25] Wang N. Peng L. Gu Y. Liang C. Pott R. W. M. Xu Y. (2023). Insights into biodegradation of antibiotics during the biofilm-based wastewater treatment processes. J. Clean. Prod. 393, 136321. 10.1016/j.jclepro.2023.136321

[B26] Wang Z. Chu Y. Chang H. Xie P. Zhang C. Li F. (2022). Advanced insights on removal of antibiotics by microalgae-bacteria consortia: A state-of-the-art review and emerging prospects. Chemosphere 307, 136117. 10.1016/j.chemosphere.2022.136117 35998727

[B27] Xiong Q. Hu L. X. Liu Y. S. Zhao J. L. He L. Y. Ying G. G. (2021). Microalgae-based technology for antibiotics removal: from mechanisms to application of innovational hybrid systems. Environ. Int. 155, 106594. 10.1016/j.envint.2021.106594 33940395

[B28] Yu C. Pang H. Wang J. H. Chi Z. Y. Zhang Q. Kong F. T. (2022). Occurrence of antibiotics in waters, removal by microalgae-based systems, and their toxicological effects: A review. Sci. Total Environ. 813, 151891. 10.1016/j.scitotenv.2021.151891 34826467

[B29] Zhang X. Zhao H. Du J. Qu Y. Shen C. Tan F. (2017). Occurrence, removal, and risk assessment of antibiotics in 12 wastewater treatment plants from Dalian, China. Environ. Sci. Pollut. Res. Int. 24, 16478–16487. 10.1007/s11356-017-9296-7 28551746

